# The Effect of Fluctuating Incubation Temperatures on West Nile Virus Infection in *Culex* Mosquitoes

**DOI:** 10.3390/v13091822

**Published:** 2021-09-14

**Authors:** Bethany L. McGregor, Joan L. Kenney, C. Roxanne Connelly

**Affiliations:** 1 Center for Grain and Animal Health Research, Agricultural Research Service, United States Department of Agriculture, 1515 College Ave., Manhattan, KS 66502, USA; 2 National Center for Emerging and Zoonotic Infectious Diseases, Centers for Disease Control and Prevention, Division of Vector-Borne Diseases, 3156 Rampart Road, Fort Collins, CO 80521, USA; vwx1@cdc.gov (J.L.K.); csz5@cdc.gov (C.R.C.)

**Keywords:** daily temperature range, vector competence, *Culex quinquefasciatus*, *Culex tarsalis*, West Nile virus

## Abstract

Temperature plays a significant role in the vector competence, extrinsic incubation period, and intensity of infection of arboviruses within mosquito vectors. Most laboratory infection studies use static incubation temperatures that may not accurately reflect daily temperature ranges (DTR) to which mosquitoes are exposed. This could potentially compromise the application of results to real world scenarios. We evaluated the effect of fluctuating DTR versus static temperature treatments on the infection, dissemination, and transmission rates and viral titers of *Culex tarsalis* and *Culex quinquefasciatus* mosquitoes for West Nile virus. Two DTR regimens were tested including an 11 and 15 °C range, both fluctuating around an average temperature of 28 °C. Overall, no significant differences were found between DTR and static treatments for infection, dissemination, or transmission rates for either species. However, significant treatment differences were identified for both *Cx. tarsalis* and *Cx. quinquefasciatus* viral titers. These effects were species-specific and most prominent later in the infection. These results indicate that future studies on WNV infections in *Culex* mosquitoes should consider employing realistic DTRs to reflect interactions most accurately between the virus, vector, and environment.

## 1. Introduction

The epidemiology of vector-borne disease is inherently complex, involving multifaceted interactions between vectors, hosts, and pathogens occurring within a constantly changing environment. Numerous environmental variables impact transmission dynamics, but perhaps none greater than temperature [1]. Temperature is a factor in the calculation of metrics associated with the vector potential of an organism or population, including the extrinsic incubation period and vectorial capacity [2,3]. Aspects of vector biology, such as development time, body size, fecundity, and survival rates, are also closely associated with temperature [4,5,6,7,8]. Additionally, there is increasing evidence that gene expression of functions such as metabolism, immunity, and detoxification have a circadian component in mosquitoes [9], with light and temperature often being drivers of circadian rhythms [10]. Due to this close association between temperature and vector-borne disease, climate change is anticipated to shift the range of vectors and vector-borne diseases in the future [11,12,13,14]. Consequently, variations in temperatures used for laboratory vector competence assays have implications for real-world applications of results.

Vector competence studies investigate whether a suspected vector species can become infected with and later transmit a pathogen [15,16]. Most vector competence assays are conducted at a static temperature representing conditions expected within a region of interest [17,18,19]. However, these static temperature trials do not take into consideration circadian cycles that an insect vector would experience in nature [20,21]. Previously conducted experiments using daily temperature ranges (DTRs) rather than static temperatures reveal the significant impacts that these cycles can have. *Aedes aegypti* infected with dengue virus (DENV) serotypes 1 or 2 and held in a large DTR (20 °C) setting were found to have decreased rates of infection compared to those in a small DTR (10 °C) setting [22]. Additional studies from this system also revealed a significant effect of the mean temperature around which the DTR was fluctuating [23]. Similarly, fluctuating temperatures have been shown to impact *Plasmodium* infection in *Anopheles stephensi*, with variable outcomes depending on mean temperature [24].

Since its introduction in 1999, West Nile virus (WNV) has become the most prevalent mosquito-borne disease in the United States, with cases occurring in the continental US annually [25]. The United States is composed of a broad diversity of unique regions with variable average temperatures and DTRs, many of which report annual WNV activity. For example, the Mojave and Sonoran deserts of the Southwestern United States are home to Las Vegas, Nevada and Phoenix, Arizona-cities that experienced WNV outbreaks in 2019 (37 and 153 human cases, respectively [25]). These cities experience broad daily temperature fluctuations of approximately 13 °C during peak WNV transmission periods of July-September [26]. Similar temperature fluctuations are seen in other Southern cities of the United States, such as Houston, Texas where average daily temperatures experience 11 °C fluctuations. Despite significant daily temperature ramps and evidence that temperature significantly impacts transmission of WNV [27], we have little data on the impact of these circadian patterns on *Culex* vector competence and viral load for WNV. The purpose of this study was to compare the infection, dissemination, and transmission rates and viral titers of WNV in two mosquito vector species that occur in these regions, *Cx. quinquefasciatus* and *Cx. tarsalis*, when exposed to DTR conditions reflecting these southern US localities.

## 2. Materials and Methods

### 2.1. Study Insects

Two mosquito species were reared for these experiments: *Culex quinquefasciatus* and *Culex tarsalis*. The *Cx. quinquefasciatus* came from the Sebring colony originally colonized in 1988 from a population in Florida. The *Cx. tarsalis* were from the Bakersfield colony established in 1952 from a population present in Bakersfield, CA. Both species were reared in incubators held at 27.5 °C on a 12:12 L:D circadian cycle and provided 10% sugar solution ad libitum. On the day prior to infections, mosquitoes were transferred into 16 oz paper cups covered with a double layer of mesh and moved into the BSL-3 laboratory to acclimate to the new incubators, temperature regimens, and collection cups for 24 h. Mosquitoes were provided clean water only in the 24 h prior to blood feeding to improve blood feeding success.

### 2.2. Fluctuating DTR Conditions

**Figure 1:**
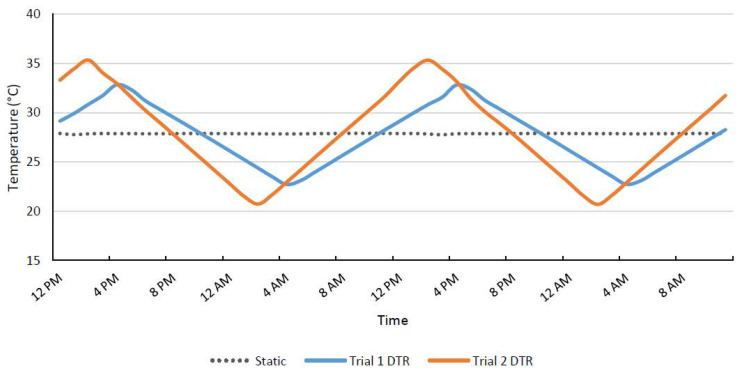
Temperature readouts of Hobo monitors placed inside incubators held at a static temperature of 28 °C, trial 1 11 °C DTR between 22.5 and 33.5 °C, and trial 2 15 °C DTR between 20.5 and 35.5 °C.

Two temperature trials were completed in which one incubator was set to oscillate temperatures gradually between a high and low temperature within a 24 h period (DTR treatment) and a second incubator was set to hold a stable temperature. Both incubators were Thermo Scientific Forma Environmental Chambers (Thermo Fisher Scientific, Waltham, MA, USA), and the DTR treatment incubator was also fitted with a temperature controller (Watlow Electric Manufacturing Co., St. Louis, MO, USA). The first trial used a DTR of 11 °C from 22.5 to 33.5 °C in one incubator (increasing at a rate of 0.015 °C/min for 12 h followed by a decrease of 0.015 °C/min for 12 h) and a static temperature of 28 °C in the other incubator (Figure 1). For the second trial, a DTR of 15 °C (20.5–35.5 °C) was used in the fluctuating incubator (increasing at a rate of 0.021 °C/min for 12 h followed by decreasing at 0.021 °C/min for 12 h) and the static incubator was set to 28 °C. Hobo monitors (Onset Computer Corporation, Bourne, MA, USA) were used throughout the experiment to monitor temperatures in both incubators.

### 2.3. Infection Trials

The viral strain of WNV used for this study was isolated from mosquitoes collected in Fort Collins, CO, USA in 2016. The stock virus was passaged twice on Vero cells and then mixed with defibrinated goose blood (Colorado Serum Company, Denver, CO, USA) to produce a final infectious blood titer of 7.0 log_10_ PFU/mL for both blood feedings in trial one and for the *Cx. quinquefasciatus* in trial 2. In trial 2, the infectious titer fed to *Cx. tarsalis* was reduced to 6.5 log_10_ PFU/mL in response to high infection rates observed in trial 1. Mosquitoes were fed infectious blood through a Parafilm membrane using a Hemotek blood-feeding system (Hemotek Ltd., Blackburn, UK) set to warm the blood to 37 °C. Mosquitoes were left to blood feed inside an incubator set at 28 °C for one hour. At the end of the blood feeding period, cups of mosquitoes were anesthetized by exposure to −20 °C for 45 s followed by placing anesthetized mosquitoes in a petri dish on ice. Blood fed mosquitoes were sorted into fresh 16 oz cups of <50 individuals while unfed and male mosquitoes were discarded.

Mosquitoes were monitored for mortality and administered fresh 10% sugar solution daily. Collections were made from both treatments and both species on days 4 and 8 post infection. An additional timepoint was added for *Cx. quinquefasciatus* at 12 days post infection in trial 2, but this timepoint was not added for *Cx. tarsalis* due to poor survival. Prior to collections, mosquitoes were anesthetized with triethylamine (TEA) (Thermo Fisher Scientific). Legs were dissected from each individual and collected into 2 mL microcentrifuge tubes containing 500 µL complete media (Dulbecco’s Modified Eagle Medium (Gibco, Thermo Fisher Scientific) supplemented with 10% fetal bovine serum (Omega Scientific Inc, Tarzana, CA, USA), 2% penicillin streptomycin solution (Gibco, Thermo Fisher Scientific), and 0.2% Amphotericin B solution (Gibco, Thermo Fisher Scientific)) and 4–8 2 mm zirconium oxide beads (Glen Mills Inc., Clifton, NJ, USA). Capillary assays were then conducted to collect mosquito saliva [28]. Capillary tubes were collected into 2 mL microcentrifuge tubes containing 300 µL complete media. Mosquito bodies were then collected into 2 mL microcentrifuge tubes containing 500 µL complete media and 4–8 2 mm zirconium oxide beads.

### 2.4. Sample Testing

Samples were homogenized and tested for virus using established protocols [29]. Plaque assays were conducted on Vero cells for all body samples. Legs were then tested for individuals with positive bodies followed by plaque assays on saliva samples for those individuals with positive legs. Serial dilutions in complete media were used when necessary to titrate samples for plaque assays.

### 2.5. Statistical Analyses

Infection rates were calculated as the total number of individuals with positive bodies divided by the total number of individuals that successfully blood fed on the infectious blood meal. Dissemination rates were calculated as the total number of individuals with positive legs divided by the total number of individuals with positive bodies. Transmission rates were calculated as the total number of individuals with positive saliva divided by the total number with positive bodies.

Infection, dissemination, and transmission rates were assessed by Fisher’s exact tests for both species and trials. Shapiro–Wilk tests were used to determine whether virus titer data followed a normal distribution. Titer data were determined to be non-normally distributed, so Wilcoxon–Mann–Whitney non-parametric tests were used to assess significant differences in viral titers between treatments in both experiments and for both species. Fisher’s exact tests and Wilcoxon–Mann–Whitney tests were also used to assess differences in the infection, dissemination, and transmission rates and viral titers between DTRs for *Cx. quinquefasciatus*.

## 3. Results

### 3.1. Trial 1: 11 °C DTR

**Figure 2:**
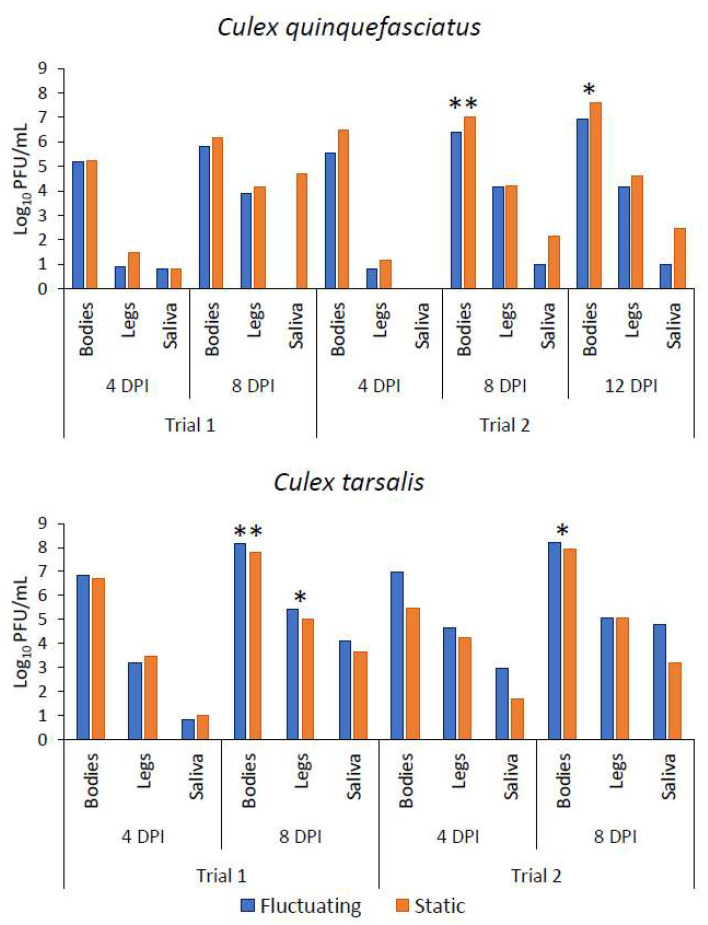
Mean viral titer in bodies, legs, and saliva of *Cx. quinquefasciatus* and *Cx. tarsalis* exposed to fluctuating and static temperature treatments at 11 °C DTR (Trial 1) and 15 °C DTR (Trial 2). Overall, *Cx. quinquefasciatus* had higher titers in static treatments in both trials while *Cx. tarsalis* had higher mean titers in fluctuating treatments. Asterisks denote significance at *p* = 0.05 (*) or *p* < 0.001 (**).

There were no significant differences identified in infection, dissemination, or transmission rates or in viral titers between treatments for *Cx. quinquefasciatus* in trial 1 at the 4 or 8 days post-infection (DPI) timepoint (Table 1 and Table 2). Too few *Cx. quinquefasciatus* individuals had positive saliva to produce a robust statistical analysis on salivary titers. *Culex quinquefasciatus* viral titers were higher overall in the static treatment than in the fluctuating treatment in trial 1 (Figure 2).

For *Cx. tarsalis*, Fisher’s exact results indicated that there was no difference between treatments in infection rates of bodies at 4 or 8 DPI (Table 1). There was also no statistical difference between titers of body samples at 4 DPI; however, a significant difference between body titers at 8 DPI was observed (Table 2). There were no differences between the two treatments in dissemination rates at either timepoint (Table 1). At day 4 post infection, there was no difference in viral titers of legs; however, a significant difference was identified in leg viral titers between treatments at 8 DPI (Table 2). No difference was identified in transmission rates between the two treatments at either timepoint (Table 1). There were too few positive saliva samples to run a robust statistical analysis on salivary titer results for 4 DPI. At 8 DPI, there was no difference identified in salivary titers between treatments (Table 2). Overall, viral titers for *Cx. tarsalis* were higher in the fluctuating group than the static group except for the 4 DPI legs and saliva (Figure 2).

### 3.2. Trial 2: 15 °C DTR

No treatment effect was observed for the infection, dissemination, or transmission rates of *Cx. quinquefasciatus* at any of the three timepoints for trial 2 (Table 1). At 4 DPI, no difference was observed between treatments in body titers or leg titers (Table 2). There were no positive saliva samples at 4 DPI to run titer analyses. At 8 DPI, a significantly higher viral titer was observed in body tissues of insects exposed to static temperatures. No difference was observed at 8 DPI for leg titers or saliva titers (Table 2). Similarly, at 12 DPI, significantly higher viral titers were observed in the body tissues of individuals in the static treatment (Table 2). Leg titers and saliva titers again showed no difference between treatments (Table 2). Similar to trial 1, *Cx. quinquefasciatus* had higher viral titers overall in the static treatment (Figure 2).

There was no treatment effect identified for *Cx. tarsalis* for infection, dissemination, or transmission rates (Table 1). No difference was identified in body titers or leg titers at 4 DPI, with too few positive saliva samples to run statistical tests. At 8 DPI, body titers were significantly higher in the fluctuating treatment group; however, leg titers and saliva titers were not statistically different at 8 DPI for *Cx. tarsalis* (Table 2). The overall titers for *Cx. tarsalis* were higher in the fluctuating group than in the static group in trial 2 except in the 8 DPI legs (Figure 2).

### 3.3. Trial Comparison—Culex quinquefasciatus

**Figure 3:**
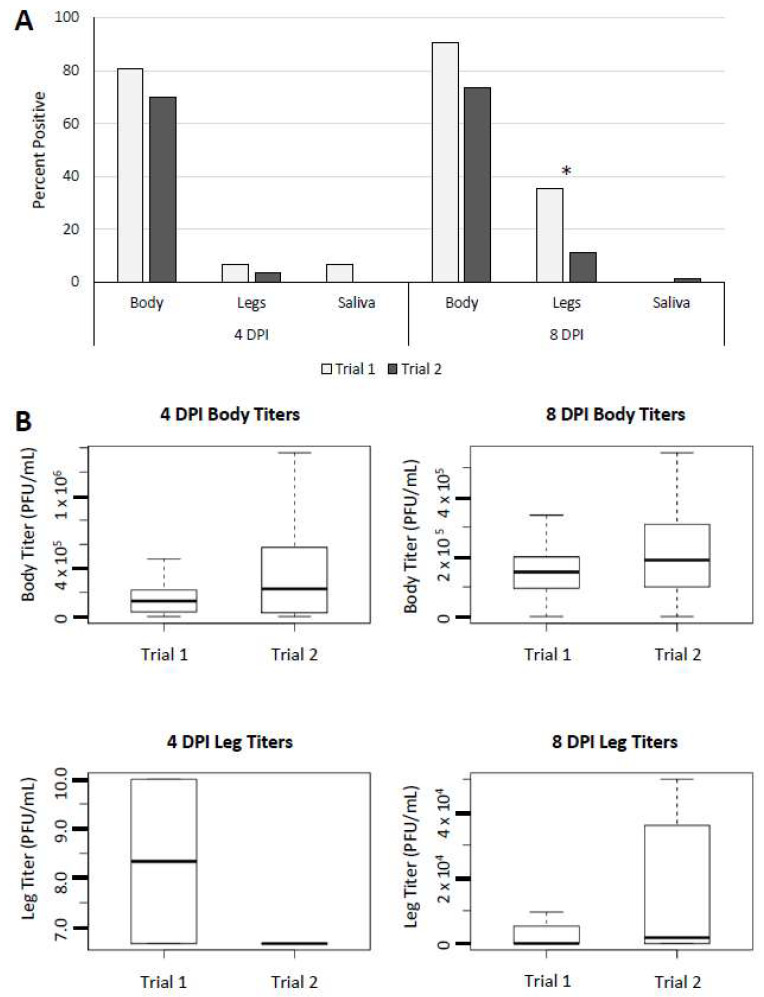
Comparison of *Culex quinquefasciatus* outcomes when exposed to DTRs of 11 °C (Trial 1) and 15 °C (Trial 2). (**A**) Overall percent positive bodies (infection), legs (dissemination), and saliva (transmission potential); Significance at *p* = 0.05 is denoted with *. (**B**) Box plots showing comparative body and leg titers at 4 DPI (on left) and 8 DPI (on right).

*Culex quinquefasciatus* was exposed to the same viral titer during each trial, permitting statistical comparison of outcomes between large and small DTRs at 4 and 8 DPI. At 4 DPI, outcomes in bodies, legs, and saliva were not different between the 11 and 15 °C DTR trials. Body and saliva outcomes were also not statistically different between fluctuation treatments at 8 DPI; however, there was a significant difference in leg infections at 8 DPI (*p* = 0.02) with 39.3% of individuals with a positive body also showing dissemination to legs in trial 1 versus 15.2% of individuals in trial 2 (Figure 3A). There were no differences identified between trials in body titers at 4 DPI (*p* = 0.06) or 8 DPI (*p* = 0.42) or in leg titers at either timepoint (4 DPI *p* = 0.41, 8 DPI *p* = 0.29) (Figure 3B).

## 4. Discussion

We tested whether *Cx. quinquefasciatus* and *Cx. tarsalis* fed blood containing WNV would show variable vector competence when exposed to either static or fluctuating incubation temperatures. The results of this study suggest that while infection, dissemination, and transmission rates appear to be comparable between treatments, viral titers may be more susceptible to changes in temperature treatment. Interestingly, these results appear to be species specific. *Culex quinquefasciatus* had higher viral titers in static treatments overall while *Cx. tarsalis* generally had higher viral titers in fluctuating treatments.

It is unclear what led to these species-specific responses to different temperature treatments, although this phenomenon has been seen in similar studies conducted with *Aedes aegypti* and *Aedes albopictus* exposed to Chikungunya virus [21]. It is possible that the physiology of infection differs between these species and between exposure temperatures. While WNV specific tissue tropisms have been investigated in *Cx. quinquefasciatus* [30], less information is available for *Cx. tarsalis* to permit direct comparisons. In general, previous evaluations of these two species have shown higher vector competence of *Cx. tarsalis* populations for WNV than *Cx. quinquefasciatus* [31]. Higher temperatures have been shown to result in higher titer infections and shortened extrinsic incubation periods in *Cx. tarsalis*, although this relationship may be dependent on viral strain [32]. Overall, the periods of high temperature in the fluctuating treatment may have had a disproportionate effect on the viral titer of body tissues in this species to the minimum temperatures experienced [33]. Furthermore, this phenomenon could be related to differences in the geographic range of the two species. The range of *Cx. tarsalis* extends much further north than that of *Cx. quinquefasciatus* [27]. This broader range into more northerly areas may result in different physiological responses to temperature changes in this species.

For *Cx. quinquefasciatus*, there appeared to be very little difference in outcomes between the two DTR treatments except for dissemination rates. At 8 DPI, significantly more *Cx. quinquefasciatus* displayed a disseminated infection in the 11 °C treatment compared with the 15 °C DTR treatment. Considering the lack of significant differences between fluctuating and static treatments, this was a surprising outcome. There is some evidence that at high temperatures of 32 °C, female *Cx. tarsalis* show some capacity to modulate and prevent dissemination of western equine encephalomyelitis virus compared with those incubated at lower temperatures of 18 and 25 °C [34]. If this is also the case in *Cx. quinquefasciatus* with WNV, periods of high temperatures associated with the high DTR treatment may have reduced dissemination overall. Mosquitoes in the high DTR treatment were exposed to temperatures >32 °C for around 5 h compared to under 2 h in the low DTR treatment.

These results are increasingly relevant in light of ongoing discussions of climate change impacts on global disease transmission dynamics. In general, climate change in North America is anticipated to result in elevated temperatures, shorter winters, and increasing extreme weather events including heavy rainfall—all of which have the potential to impact vector-borne disease dynamics [35]. Additionally, DTR’s are anticipated to shift in response to climate change with some models indicating that minimum daily temperatures are likely to increase more than maximum daily temperatures on average, potentially leading to smaller DTRs [36,37]. This makes our finding of significantly higher dissemination in *Cx. quinquefasciatus* at the smaller DTR more concerning. This also further emphasizes the need for research on impacts of climate change on vector-borne diseases.

The present study included some challenges and limitations that must be acknowledged. The first limitation was the age of the two mosquito colonies used for these experiments. The colonies used are well characterized, stable laboratory populations that can provide good baseline data; however, younger colonies would have been useful to understand how populations acclimated to temperature fluctuations responded to our treatments. Attempts to establish young colonies for these experiments to run alongside colony specimens unfortunately failed. Additional studies are encouraged to investigate these patterns in young colonies or field populations that may be more adapted to temperature fluctuations. Additionally, the DTR regimens used in these trials did not follow typical sinusoidal temperature patterns due to software limitations in the temperature controllers. While the software allowed us to program smooth, constant temperature ramping, it was not a perfect reflection of natural temperature fluctuations. We also only tested fluctuations occurring around a single mean temperature. Studies conducted on other systems have found that the magnitude and direction of effects of fluctuating temperatures may depend on the mean temperature being studied [23,24]. Further studies on the impact of fluctuations around additional mean temperatures are warranted. Finally, both *Cx. tarsalis* and *Cx. quinquefasciatus* are considered crepuscular to nocturnal [38,39,40,41] and are likely to be in sheltered locations during periods of peak heat. The temperature regimens we used replicated those that would be anticipated in areas such as Houston, TX or Las Vegas, NV during WNV outbreaks and were modelled after temperature records from these locations. Most temperature records are taken from exposed areas rather than sheltered locations, so the high temperatures of our fluctuating treatments may have been hotter than these mosquitoes would have experienced naturally.

## 5. Conclusions

These experiments provide evidence that virus titers in the bodies, and in some cases the legs, of *Cx. quinquefasciatus* and *Cx. tarsalis* can be significantly different between fluctuating and static temperature conditions. For studies investigating virus titer dynamics in arthropod hosts, replicating natural temperature fluctuations may be advantageous to get the most accurate representation of viral titers over time. However, for studies investigating infection, dissemination, or transmission rates, our results indicated that temperature fluctuations did not meaningfully impact these values and average, static temperatures may be adequate. These results are likely species specific and may need to be investigated on a case-by-case basis.

## Figures and Tables

**Table 1:** Positive bodies (infection), legs (dissemination), and saliva (transmission potential) for *Culex quinquefasciatus* and *Culex tarsalis* exposed to static or fluctuating incubation treatments. No significant differences were observed in infection, dissemination, or transmission rates between temperature treatments for either species or trial.

*Culex* Species	Trial	Time (DPI)	Treatment	Bodies ^†^	Bodies *p*	Legs ^†^	Legs *p*	Saliva ^†^	Saliva *p*
*quinquefasciatus*	1	4	Static	29/31	0.255	2/29	1	1/2	1
			Fluctuating	25/31		2/25		2/2	
		8	Static	28/32	1	9/28	0.781	1/9	0.450
			Fluctuating	28/31		11/28		0/11	
	2	4	Static	69/89	0.319	4/69	1	0/4	1
			Fluctuating	63/90		3/63		0/3	
		8	Static	62/90	0.622	10/62	0.622	2/10	1
			Fluctuating	66/90		10/66		1/10	
		12	Static	76/113	0.241	39/76	1	10/39	0.219
			Fluctuating	51/87		25/51		2/25	
*tarsalis*	1	4	Static	19/23	0.109	11/19	0.213	4/11	0.338
			Fluctuating	23/23		8/23		1/8	
		8	Static	24/25	1	22/24	0.489	20/22	0.223
			Fluctuating	24/26		24/24		24/24	
	2	4	Static	42/63	0.714	7/42	0.757	1/7	1
			Fluctuating	41/65		5/41		1/5	
		8	Static	19/41	0.691	15/19	0.456	10/15	0.716
			Fluctuating	28/68		25/28		19/25	

^†^ Bodies: positive bodies/total bodies tested; Legs: positive legs/positive bodies; Saliva: positive saliva/positive bodies.

**Table 2:** Average viral titer present in bodies, legs, and saliva of *Culex quinquefasciatus* and *Culex tarsalis* in trial 1 (11 °C DTR) and trial 2 (15 °C DTR). All viral titers are expressed in log_10_ PFU/mL.

*Culex* Species	Trial	Time (DPI)	Treatment	Body Titer	Body *p*	Leg Titer	Leg *p*	Saliva Titer	Saliva *p*
*quinquefasciatus*	1	4	Static	5.24	0.574	1.48	0.333	0.82	n/a
			Fluctuating	5.20		0.92		0.82	
		8	Static	6.18	0.054	4.17	0.619	4.69	n/a
			Fluctuating	5.82		3.90		n/a	
	2	4	Static	6.49	0.421	1.18	0.270	n/a	n/a
			Fluctuating	5.55		0.82		n/a	
		8	Static	7.02	**<0.001**	4.20	0.791	2.15	0.667
			Fluctuating	6.39		4.17		1.00	
		12	Static	7.62	**0.007**	4.60	0.821	2.49	0.075
			Fluctuating	6.92		4.18		1.00	
*tarsalis*	1	4	Static	6.71	0.062	3.47	0.342	1.00	n/a
			Fluctuating	6.84		3.19		0.82	
		8	Static	7.81	**<0.001**	5.01	**0.008**	3.67	0.191
			Fluctuating	8.15		5.44		4.11	
	2	4	Static	5.46	0.068	4.25	0.222	1.70	n/a
			Fluctuating	6.97		4.66		2.99	
		8	Static	7.95	**0.025**	5.07	0.665	3.18	0.854
			Fluctuating	8.23		5.06		4.79	

Significant *p*-values are shown in bold.

## Data Availability

Data are available from the authors by request.
